# Enablers and barriers to evidence based planning in the district health system in Uganda; perceptions of district health managers

**DOI:** 10.1186/s12913-017-2059-9

**Published:** 2017-02-02

**Authors:** Dorcus Kiwanuka Henriksson, Florence Ayebare, Peter Waiswa, Stefan Swartling Peterson, Elly K. Tumushabe, Mio Fredriksson

**Affiliations:** 1 Karolinska Institutet, Stockholm, Sweden; 2 Uppsala University, Uppsala, Sweden; 3 Makerere University College of Health Sciences, School of Public Health, Kampala, Uganda; 4 Mukono District Local Government, Mukono, Uganda; 5 Department of Public Health and Caring Sciences, Uppsala University, Uppsala, Sweden

**Keywords:** District, Planning, Health systems, Evidence, Managers, Decentralization, Politicians, Uganda

## Abstract

**Background:**

The District Health System was endorsed as the key strategy to achieve ‘Health for all’ during the WHO organized inter-regional meeting in Harare in 1987. Many expectations were put upon the district health system, including planning. Although planning should be evidence based to prioritize activities, in Uganda it has been described as occurring more by chance than by choice. The role of planning is entrusted to the district health managers with support from the Ministry of Health and other stakeholders, but there is limited knowledge on the district health manager’s capacity to carry out evidence-based planning. The aim of this study was to determine the barriers and enablers to evidence-based planning at the district level.

**Methods:**

This qualitative study collected data through key informant interviews with district managers from two purposefully selected districts in Uganda that have been implementing evidence-based planning. A deductive process of thematic analysis was used to classify responses within themes.

**Results:**

There were considerable differences between the districts in regard to the barriers and enablers for evidence-based planning. Variations could be attributed to specific contextual and environmental differences such as human resource levels, date of establishment of the district, funding and the sociopolitical environment. The perceived lack of local decision space coupled with the perception that the politicians had all the power while having limited knowledge on evidence-based planning was considered an important barrier.

**Conclusion:**

There is a need to review the mandate of the district managers to make decisions in the planning process and the range of decision space available within the district health system. Given the important role elected officials play in a decentralized system a concerted effort should be made to increase their knowledge on evidence-based planning and the district health system as a whole.

## Background

The District Health System (DHS) received political endorsement as the key strategy to achieve ‘Health for all’ during the WHO organized inter-regional meeting in Harare, Zimbabwe in 1987 [1]. Since then health systems in many African countries have undergone considerable reforms with decentralization of health services being central to these changes [2, 3]. These reforms were intended to promote more accountability by the district health system, local preference [4], community participation [1] and to make health systems more equitable, inclusive and fair [5]. In Uganda authority was transferred from the central government to the local government authorities in 1997, mainly in the form of devolution [6, 7], which refers to the shift of authority, responsibility and accountability from the central government to lower autonomous entities, provincial or municipal governments [2, 8, 9]. Unlike many other countries, Uganda has no functional “intermediate level” for example a province or region [10].

In Uganda, the District Health System is headed by appointed officials, the District Health Team (DHT) in collaboration with the wider District Health Management Team (DHMT) both headed by the District Health Officer (DHO) [10] and governed by a district council of elected officials [6, 11]. After the Harare Declaration, many expectations were put upon the district health system; planning, health data analysis, budgeting, allocation of resources, leadership, co-ordination of response to emergencies, supervision and training [1]. Planning is one of the key functions of the district health managers and central to the performance of the health system [12]. While the Ugandan health system is decentralized, most of the priority setting is carried out at the national level and districts follow the national guidelines [13]. Although planning should increasingly be evidence based to prioritize activities [14, 15], priority setting in Low and Middle-Income Countries (LMIC) like Uganda has been described as ad-hoc [13] and seldom evidence-based [16]. Evidence-based planning (EBP) is the process of basing decisions about ways to address a problem on objective information in order to achieve the best results [17]. Other studies showed that priority setting in the planning process was in the context of budget cycles and driven by historical allocation of funds and not necessarily by evidence [16, 18] and others have documented the political and technical resistance to decentralization and the limited operational responsibility of the DHMT as being an influence to the district planning process [19, 20]. Donor and other institution priorities and concerns for example about measurable results and promotion of disease specific programs has also had an effect on the district planning process [21, 22]. In Uganda, it has been described as occurring more by chance than by choice [23] with performance discrepancies reported across and within districts [24]. For example, this has led the Ministry of Health (MoH) in Uganda to initiate a critical review and reflection of the DHS strategy [21].

The role of planning is entrusted to the district health managers and is based on district planning meetings involving the MoH staff, district staff and other stakeholders [10, 21]. There is currently limited knowledge on the district health manager’s capacity to carry out evidence-based planning in this context. For instance, are the district health managers empowered and able to spearhead planning of effective, efficient and quality service delivery? Furthermore, what happens in the intersection between the technical and the political decision makers? To answer these questions it is important to study and determine the barriers and enablers to the evidence-based planning process at the district level, which was the aim of this study.

A set of theoretical domains [25] that have previously been used to assess barriers and enablers to delivery of the Healthy Kid Checks [26], to implementing antenatal magnesium sulphate for fetal neuroprotection guidelines [27], careful hand hygiene as perceived by nurses and hospital administrators [28] and preconception care guidelines [29] were used in this study (See Table 1). The theoretical domains go beyond the evidence-based planning process itself and examine the context and environment within which it is taking place and the people involved which is in keeping with the complex nature of the district health system.

**Table 1:** Theoretical Domains Framework and constructs

Theoretical domains	Constructs
Knowledge	Knowledge about planning Knowledge about evidence-based planning (EBP) Procedural knowledge
Skills	Competence/ability Skills development Interpersonal skills Coping strategies
Social and professional roles (Self-standards)	Professional identity/role Group /social identity Social/group norms Organizational commitment
Beliefs about capability (Self- efficacy)	Self-efficacy Control of behavior, material and social environment Perceived competence Self/professional confidence Empowerment Optimism/pessimism
Beliefs about consequences (Anticipated outcomes/attitudes)	Outcome expectancies Consequences Attitudes Incentives/rewards/sanctions Beliefs Characteristics of outcome expectations – physical, social, emotional Valued/not valued Perceived risk/threat
Motivation and goals (Intentions)	Intention; stability of intention/certainty of intention Goals (autonomous, controlled) Goal target/setting Goal priority Intrinsic motivation Commitment
Memory, attention and decision process	Memory Attention Decision making
Environmental context and resources (Environmental constraints)	Resources/material resources (availability and management) Environmental stressors Person and environmental interaction Knowledge of task environment
Social influence (Norms)	Social support; personal/professional/organizational/society/community Social/group norms Leadership Team work Organizational climate/climate Social pressure Power/hierarchy Professional boundaries/roles Supervision Inter-group conflict Conflict- competing demands, conflicting roles

## Methods

### The CODES project

This study was conducted in two districts in Uganda; districts that have implemented the Community and District Empowerment for Scale-Up (CODES) project funded by the Bill & Melinda Gates Foundation [30, 31] for over two years. The project uses local data to analyze bottlenecks in order to systematize priority setting, allocation of resources and problem-solving as a strategy to facilitate evidence-based planning [32]. This is the approach or strategy being implemented in 13 districts in Uganda under the CODES project which works within the district planning cycle and focuses on scaling up child survival interventions by identifying bottlenecks that constrain provision and access to care and determining which set of evidence-based strategies are most likely to increase coverage [31]. The project introduces district managers to the tools that can enable the EBP process and builds their capacity in being able to use these tools and adapt them to the district planning cycle [30, 32].

### Study design

This was a qualitative study. This design was used because it allows DHMT members to freely discuss the planning process and have in-depth discussions [33, 34] about the barriers and enablers.

### Study sites and selection criteria

**Fig. 1:**
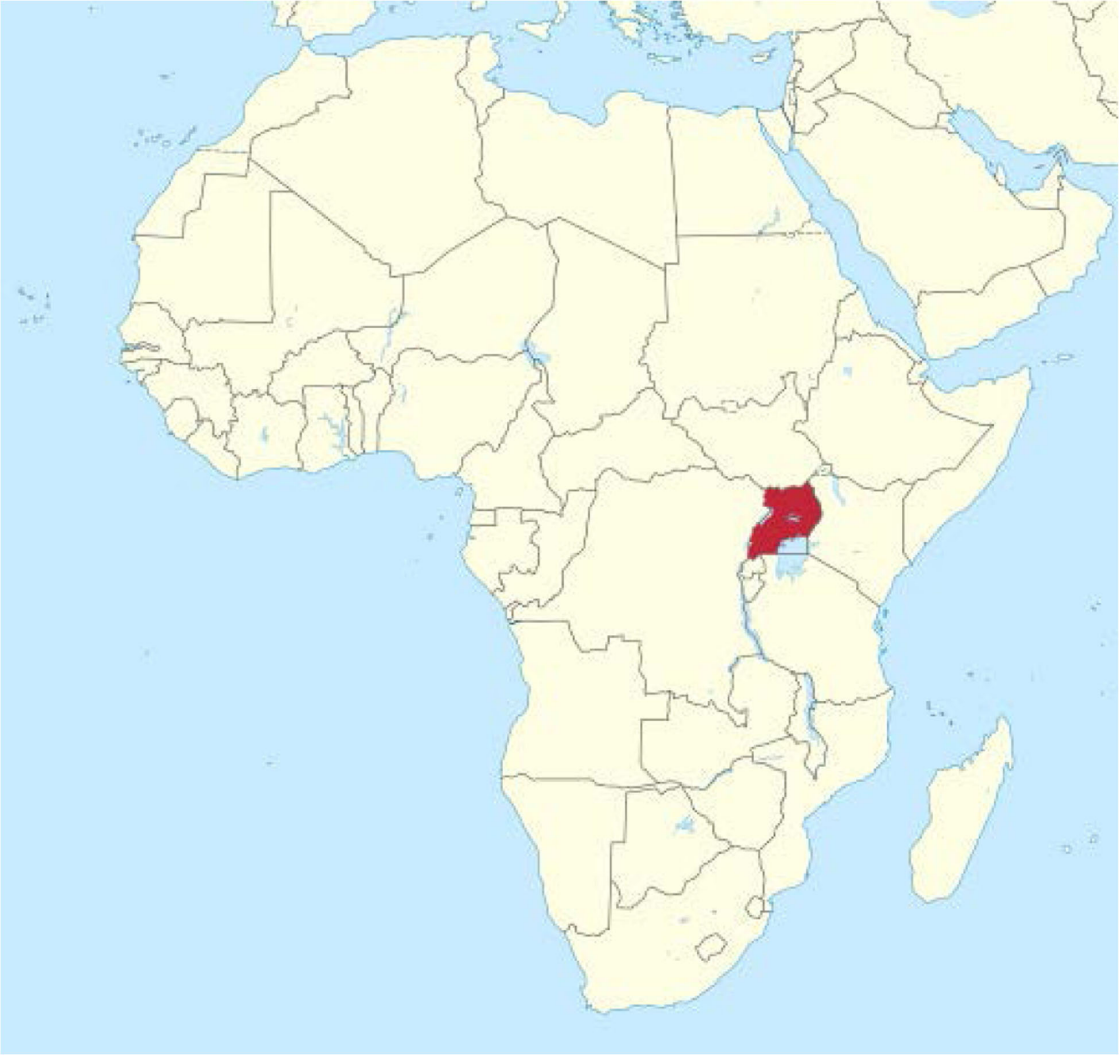
Map of Africa showing the location of Uganda. ©CC BY-SA 3.0 TUBS; Wikimedia Commons

This study was conducted in two purposively selected districts in Uganda (See Fig. 1). The districts were selected because the study assumed that given the two districts’ participation in the CODES project the DHMT members would be likely to contribute with relevant and well-founded information on barriers and enablers for district managers to carry out EBP. One of the districts included in this study was established in the 1990s and for the purpose of this study referred to as district A. The second district was established in 2010 and for the purpose of this study referred to as district B. Both districts are mainly rural with approximately 75% of the population living in rural areas (Table 2) and have agriculture as the main economic activity. Approximately 58% of the population in both districts is 18 years and below.

**Table 2:** District demographics

Demographics	District A	District B
Approximate^a^ total population	300,000	150,000
Year of creation	1990	2010
Approximate rural population	200,000	140,000
Approximate urban population	100,000	10,000
Number of health facilities	20	14

^a^Approximates were used to keep districts anonymous

### Study participants and sample size

Sixteen participants were included in this study and these were DHMT members from the two districts. They were purposively selected [35] because of their knowledge, involvement, and different functions in the planning process and represented a variety of perspectives.

### Recruitment of participants and consent

Participants were invited to take part in the study through the DHO’s office. After the participants agreed to take part, telephone calls were made to each of the participants by the 1st and 2nd author and appointments were made for the face - to - face interviews. More information about the study was given, which included anonymity of participants, the fact that they were not getting paid for the interviews and their participation was voluntary. Individual verbal informed consent was obtained from the participants at the beginning of the interview.

### Study team

The research team consisted of two Ugandan Public health specialists (DKH) and (PW) with experience as heads of a DHT, a Ugandan research assistant (FA), a Swedish health systems specialist (SSP) with previous experience working in Uganda and a Swedish researcher in health and political reforms (MF). No one on the team was working within the district health system.

### Data collection and procedure

Data was collected through semi-structured interviews conducted by the 1^st^ and 2^nd^ author in March 2015 with members of the DHMT as key informants [36]. Fifteen of the interviews were conducted in English and one in Luganda (local language) by the 1^st^ author. Interviews were audio recorded apart from one where the participant declined being recorded. Each interview lasted approximately 60 min apart from one that lasted 90 min. All interviews were transcribed verbatim including the one that was conducted in Luganda, which was first translated into English and then transcribed.

### Interview guide

An interview guide based on the theoretical domain framework [25] was developed and pre-tested by the first author with DHMT members from districts not included in the study. The framework outlines 12 key theoretical domains that are most likely to best explain implementation problems [25, 26]. After pre-testing, nine out of the 12 domains were included in this study; knowledge, skills, social and professional roles, beliefs about capability, beliefs about consequences, motivation, and goals, memory, attention and decision process, environmental context and resources and social influences [25] as shown in Table 1. The three domains, emotion, behavior regulation and nature of the behaviors were not used in this study because they generated little or no information during the pre-testing of the interview guide.

### Data analysis

The first and last author independently read through two transcripts, coded the data separately and came together to discuss and establish a consensus on coding. This was to establish a common meaning and understanding of the data and try to address the subjectivity of the 1^st^ author who has previously been a DHO. After agreement had been reached on the coding process the first author then read and reread the entire dataset and coded the data from each interview. A deductive process of thematic analysis [37–39] was used to classify responses within themes and the theoretical domains previously described were used as a coding framework. All the data collected was represented within the domains of the framework and in some instances, some of the data was allocated to more than one domain.

## Results

**Fig. 2:**
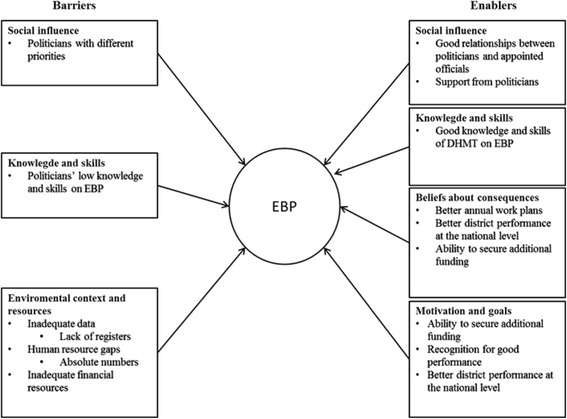
Barriers and enablers to EBP in district A

**Fig. 3:**
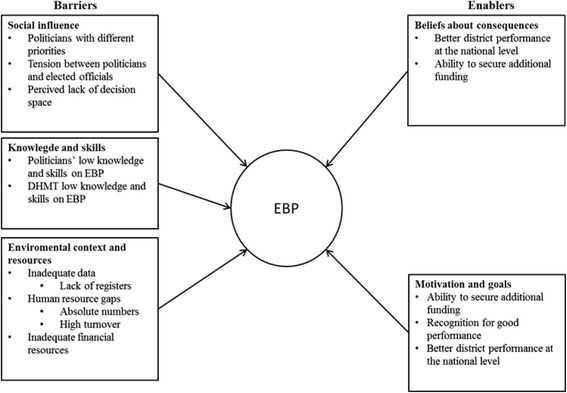
Barriers and enablers to EBP in district B

The barriers and enablers to EBP were mainly expressed in six of the domains: 1) Knowledge; 2) Skills; 3) Environmental context and resources; 4) Social influences; 5) Beliefs about consequences; and 6) Motivation and goals. However, as shown in Figs. 2 and 3, the barriers and the enablers varied between the two districts.

### Knowledge and skills

DHMT members’ from district A reported generally high levels of knowledge and skills on the EBP process as illustrated by the quote.

*“Yeah, the necessary knowledge is available because I have members of my team that have been involved during the data collection and surveys, yes.”*

In district B, DHMT members generally expressed the need for additional training or support to improve their EBP skills:

*“I am not saying am very competent, if I got extra training, I don’t want a master in planning, for example, mentorships with somebody who knows these things.”*

However, in both districts DHMT members mentioned that some of the elected officials (who are important in the overall district planning process) did not have the necessary knowledge and skills for EBP (See Figs. 2 and 3), which sometimes led to difficulties in the planning process.

*“But some of the difficulties are that the people we are dealing with lack the skills and knowledge, for example, some of the findings may also be disputed because of the ignorance, so convincing them is not easy.”*

### Environmental context and resources

The DHMT members in both districts reported the biggest barriers to EBP within the environmental context and resources domain. These included the lack of financial resources that ranged from being unable to collect data from the facilities to the lack of funds to implement planned activities. This even made some DHMT members question how useful EBP is:

*“Why go through this process (EBP) when you know that the resources needed for the planned activity are not available?”*

Gaps in human resources were mentioned as a major barrier mainly in district B although adequate staffing was also mentioned as an important resource for the evidence-based planning process. DHMT members also mentioned having competing tasks and multiple roles and yet the planning process was considered time consuming. Some, however, said that once you have gone through the process several times, it becomes less time consuming and gives better results in the planning process.

*“The most challenging bit about it (EBP) is that it is lengthy. But when you look at the output, it’s good. So if you look at the advantages (good output) and compare with what you call a disadvantage, being lengthy, I think the advantages out way”*

This was sometimes confounded by incomplete staffing levels mostly in district B and the high turnover and reshuffling of staff as illustrated by this quote:

*“…what happens with districts is that, if the In-charge today was this one and was trained in that and the next day there will be a reshuffle and somebody else is there.”*

Another important resource mentioned in both districts was locally generated data on health service delivery, although many reported gaps in this area. This was mainly due to unavailability of data collection and reporting tools e.g., registers, lack of fuel to collect data from the various health facilities, and inaccurate data reports from the facilities. Inaccuracy was mainly attributed to completing registers retrospectively due to the high workload and a lack of interest in health information systems.

*“Hmm, (silence) there are problems in data collection and mainly sometimes health facilities lack tools to collect data.”*

*“One of the problems is this; you have two nurses at the facility, they are going to treat patients, they are going to record whatever they do, so it is too much work to do, that they will not concentrate on the data. In Uganda, the practice is that data is always considered and done last if there is time, so there is no interest.*

DHMT members reported that when accurate data was readily available it was easier to reach a consensus on what activities were to be prioritized as these were supported by local evidence.

However, in both districts, it was reported that even when data was available, it was not always considered when prioritizing activities. Sometimes other stakeholders involved in the planning process, e.g., the politicians, had different priorities that were not necessarily guided by the evidence.

*“You know the politicians are not technical in using the evidence for planning, their planning depends on their priorities, even if you are doing worse or well they need what they want to be passed in your plan.”*

### Social influences

DHMT members reported that the elected politicians had all the power since they made the final decision on whether to approve the district work plan or not.

*“Because the councilors are the ruling body they tend to dictate on how resources are allocated and how we should be spending what we have, although we might advise them that this is the most pressing issue for them they could have a political idea they want, so we are usually forced into a direction of what we do not want, because they are our bosses we have to implement what they want.”*

Where there was perceived tension in the relationship between DHMT members and the politicians, this was a barrier to EBP, which was more common in district B (See Fig. 3). However, in district A, where DHMT members reported longer working relationships and trust between them and the politicians, the relationship was considered more of an enabler to EBP (See Fig. 2).

The perceived lack of decision space to carry out EBP was mentioned as a barrier mainly in district B.

*“At times when we don’t have decision space, you identify the gaps and you come up with solutions then you fail to get support for the intervention you have come up with, that is demoralizing and it is very discouraging. If I cannot address my gaps, my bottlenecks then why should I really continue (Laughs) why bother?”*

The national district league table for district health system performance assessment that yearly presents district performance against a number of input, process and output indicators and a composite index to rank districts was perceived an important social influence for EBP and was considered an enabler. In both districts, a higher ranking in the league table than the previous year was partly attributed to EBP.

### Beliefs about consequences

DHMT members believed that using locally generated data in the planning process was a better way to plan and was worthwhile because it resulted in better work plans that reflected the needs of the district and not what DHMT members wanted or considered most convenient and easy to achieve, or what was implemented the previous year, as was the common practice before. This they believed led to better performance not only in their areas of responsibility but also better performance as reflected in the national district league table for district health systems performance. This good performance led to recognition from peers from within and outside the district and made their roles more relevant and enabled them to carry out their duties better. However, the majority of DHMT members were of the view that recognition should be systematized as a way of further motivate the staff.

### Motivation and goals

In both districts, the health departments were motivated to use EBP because they stated that they were able to secure additional funding from other partners and the local government for originally unfunded priorities as a result of using local data in the planning process.

*“I went to the executive, to the council using that data I told them we have to train the staff, at least we have TOT (trainers of trainees) and I did that using the data. Using the data I was able to get the money and I saw I am doing my job”*

DHMT members in both districts expressed high levels of commitment to EBP and their intention to continue doing so as expressed in these quotes:

*“Now what can I say, evidence based planning is the way to go, because the resources are too minimal, if you do not have the figures you may not allocate the resource properly”*

*“We were planning blindly but I can’t go back to that, me I feel we should maintain this.”*

## Discussion

Findings from the study showed that barriers and enablers to EBP as perceived by the DHMT members varied between the two districts as shown in Figs. 2 and 3. DHMT members from district B, the newer district that is six years old, expressed more barriers than enablers and more barriers compared to district A, the older district that is over 20 years old. However, some of the barriers and enablers were common to both districts for example in the environmental context and resources, beliefs about consequences and motivation and goals domains as shown in Figs. 2 and 3.

The greatest barriers were expressed within the domains of environmental context and resources and social influence. These included inadequate financial resources, gaps in the human resources and data available for EBP, perceived lack of decision space, and politicians with their own priorities which led to perceived tensions between the appointed officials and politicians. Barriers were thus found in relation to data itself as well as in relation to resources to handle data and in the wider district context. Contextual differences between the districts could be an important factor that influences the EBP process. District A has existed for more than 20 years is located on a major highway and would, therefore, be considered less rural and has had a stable composition of DHT members many of whom have been in the district for more than five years. In contrast, district B has existed for only six years is more rural and many of the DHT members have been in the district for less than five years.

Decision space, which refers to the range of choice for local decision makers within a decentralized system [2, 40] and thus reflects on the district’s autonomy, was perceived as limited and a barrier to EBP; more so in district B. This, in spite of the extensive decentralization process that took place in Uganda with the intention to enhance local decision-making [7]. The perceived lack of decision space could to some extent be attributed to the interpretation and understanding of policy although it is not unique to EBP as it has been documented as negatively impacting maternal health service delivery in Ghana [41] and a shortcoming of decentralization [40]. The perceived limited decision space is in large a result of priority setting at the central level [10, 13, 21]. As it is a way of rationing health services and allocate resources [42–44], national priorities may not necessary be those of the district. Although DHT members from district A reported that they were able to set district priorities within the broader national priorities, those in district B expressed a limited decision space, which raises the question of the effectiveness of the EBP process at the district level within the context of central level priority setting.

Our results show that the autonomy of the district council to approve district work plans gives them power over resources [11, 45] and therefore more influence at the district, this has previously been documented by Assimwe and Musisi (2007) and Lubanga (1998). The relationships between the elected officials (district council) and the appointed officials (DHMT), here referred to as the “sociopolitical context” was generally considered an enabler to EBP in district A and a barrier to EBP in district B. DHMT members reported that where the relationships were perceived as positive and transparent, not only was EBP more evident, but the process was less time consuming than when there were perceived tensions between the DHMT and the politicians. Similar to findings from Allen’s (1990) work on local governments in India, we found that perceived tensions are sometimes a source of conflict and could lead to a delay in decision making that ultimately affects implementation or the resources available for certain activities [46].

As was found in other studies [47–50], our results showed that politicians sometimes had different priorities from those backed by evidence and this sometimes led to the tensions with DHMT members. Integration across an organization’s boundaries [51] and effective communication across structural boundaries within an organization [52] have previously been pointed out as important for the implementation of efforts such as EBP and for improving care. Our results point to the need for a deliberate effort to build and maintain trust between the elected and the appointed officials, thus integrating the view of politicians representing a perspective different from the more technically oriented DHMT members. Furthermore, in both districts the limited knowledge and skills of the politicians was perceived as a barrier to EBP. This is similar to findings in a study conducted by Sosnowy et al. (2013) on the factors affecting evidence-based decision making in local health departments in New York State, where they found that limited knowledge and capacity to use evidence in decision-making compromised its use and there was an expressed need for more capacity building [53]. Therefore building capacity for EBP should not be limited to only the DHMT members but also include the district council or politicians who ultimately represent the interests of the wider community.

Inadequate funding was mentioned as one of the major barriers to EBP in both districts despite the fact that these districts were provided some additional funding by the CODES project [30]. Inadequate funding has been cited both as a shortcoming of decentralization and as a barrier to health service delivery especially in LMIC like Uganda [54, 55]. At the district level, the DHMT members not only referred to the inadequate amounts but also to the timeliness and funds earmarked for certain activities that were not district priorities. On one hand, this could be a strong argument for EBP, i.e., to ensure that the limited resources are used for district specific priorities. On the other hand, it raises the question of whether EBP can lead to meaningful results in resource-limited settings. This further emphasizes the need to systematically harness resources provided outside the public sector [56] in the DHS to focus on district specific priorities.

Inadequate accurate and timely data both at the facility and DHMT level was found as a barrier for EBP. This was mainly attributed to the already heavy workload of the service providers, low staffing levels and high turnover as was shown by other studies [50, 57]. This was made worse by the fact that data collection is “manual” or paper-based and was not perceived as the primary or important responsibility of DHMT members and service providers and therefore received relatively little attention. Establishing an electronic health information system at the district level could improve availability and quality of data.

The attribution of better performance within the health sector to EBP in both districts was the biggest enabler and for this reason, the DHMT members were committed to continuing with the process despite the barriers. Although the Ministry of Health in Uganda recognizes the best performing districts annually through the league table [58], it is also important to systematize recognition for good performance at the district level in order to motivate staff.

The capacity building given by the CODES project provided the knowledge and skills for EBP and it should therefore not be assumed that the DHMT members can carry out EBP without additional capacity building. In our study, however, the level of knowledge and skills seemed adequate. The CODES project also provided financial support to each district, 10,000 USD per year [30] which could have enabled EBP although this was not explicitly mentioned by the DHMT members.

### Methodological considerations

The study was conducted in only two districts and therefore limits the generalizability of findings. However, the districts in the study have a mainly rural population, and are therefore a fair representation of many districts in Uganda, although the specific context will vary. The study did not collect any information from the central level which also influences the planning process in the DHS. Even with these limitations, the study provides insight into the enablers and barriers for EBP at the DHS level that can inform decision making about the district planning process.

## Conclusion

This study provides useful information on barriers and enablers to evidence-based planning within the district health system in Uganda. There were considerable differences between the districts in regard to the barriers and enablers for EBP which could be attributed to, specific contextual and environmental differences such as the sociopolitical environment, the human resource situation and the date of establishment of the district. The perceived lack of local decision space coupled with the perception that the politicians had all the power while having limited knowledge on EBP was considered an important barrier. There is a need to review the mandate of the DHT to make decisions in the planning process and also the range of decision space available within the DHS. Given the important role elected officials play and are perceived to play in a decentralized system; a concerted effort should be made to increase their knowledge on EBP and the district health system as a whole.

## Abbreviations

CODES: Community and District Empowerment for Scale-up
DHMT: District Health Management Team
DHO: District Health Officer
DHS: District Health System
DHT: District Health Team
EBP: Evidence-based planning
EPI: Expanded Program on Immunization
HMIS: Health Management Information Systems
LMIC: Low and middle income countries
MoH: Ministry of Health
UNCST: Uganda National council of Science and Technology
WHO: World Health Organization
